# A randomized controlled trial comparison of PTEBL and traditional teaching methods in “Stop the Bleed” training

**DOI:** 10.1186/s12909-024-05457-4

**Published:** 2024-04-26

**Authors:** Wanchen Zhao, Yangbo Cao, Liangrong Hu, Chenxiao Lu, Gaoming Liu, Matthew Gong, Jinshen He

**Affiliations:** 1 Department of Orthopaedic Surgery, The Third Xiangya Hospital of Central South University, Changsha, Hunan 410013 China; 2 QingFang Orthopaedic Hospital of Wugang City, Shaoyang, Hunan 422499 China; 3 Department of Orthopaedic Surgery, University of Pittsburgh, Pittsburgh, PA 15213 USA; 4 Xiangya Scool of Medicine, Central South University, Changsha, Hunan 410013, China

**Keywords:** Stop the Bleed, PTEBL teaching method, Traditional method, Hemostasis techniques, Teamwork skills

## Abstract

**Background:**

The Stop the Bleed (STB) training program was launched by the White House to minimize hemorrhagic deaths. Few studies focused on the STB were reported outside the United States. This study aimed to evaluate the effectiveness of a problem-, team- and evidence-based learning (PTEBL) approach to teaching, compared to traditional teaching methods currently employed in STB courses in China.

**Methods:**

This study was a parallel group, unmasked, randomised controlled trial. We included third-year medical students of a five-year training program from the Xiangya School of Medicine, Central South University who voluntarily participated in the trial. One hundred fifty-three medical students were randomized (1:1) into the PTEBL group (*n* = 77) or traditional group (*n* = 76). Every group was led by a single instructor. The instructor in the PTEBL group has experienced in educational reform. However, the instructor in the traditional group follows a traditional teaching mode. The teaching courses for both student groups had the same duration of four hours. Questionnaires were conducted to assess teaching quality before and after the course. The trial was registered in the Central South University (No. 2021JY188).

**Results:**

In the PTEBL group, students reported mastery in three fundamental STB skills—Direct Finger Compression (61/77, 79.2%), Packing (72/77, 93.8%), and Tourniquet Placement (71/77, 92.2%) respectively, while 76.3% (58/76), 89.5% (68/76), and 88.2% (67/76) of students in the traditional group (*P* > 0.05 for each pairwise comparison). 96.1% (74/77) of students in the PTEBL group felt prepared to help in an emergency, while 90.8% (69/76) of students in the traditional group (*P* > 0.05). 94.8% (73/77) of students reported improved teamwork skills after the PTEBL course, in contrast with 81.6% (62/76) of students in the traditional course (*P* = 0.011). Furthermore, a positive correlation was observed between improved clinical thinking skills and improved teamwork skills (*R* = 0.82, 95% CI: 0.74–0.88; *P* < 0.001).

**Conclusions:**

Compared with the traditional teaching method, the PTEBL method was superior in teaching teamwork skills, and has equally effectively taught hemostasis techniques in the emergency setting. The PTEBL method can be introduced to the STB training in China.

**Supplementary Information:**

The online version contains supplementary material available at 10.1186/s12909-024-05457-4.

## Introduction

According to the World Health Organization, mass traumatic injuries result in over five million deaths annually [1]. In the United States, increasing shooting incidents have contributed to this high mortality rate [2]. Due to rapid development in China, more than 700,000 motor vehicle accidents occur annually, leading to approximately 1.3 million injuries and 80,000 to 100,000 deaths [3, 4]. Traumatic hemorrhage remains a significant cause of death for all ages regardless of the form of trauma [5]. It is estimated that 57% of deaths could be avoided with proper control of bleeding [6–8]. In 2015, the White House launched the Stop the Bleed (STB) training program to minimize preventable deaths from trauma [9, 10]. Bleeding control techniques of both medical professionals and the general public have indeed improved through this campaign, with a 63% decrease in deaths from uncontrolled bleeding [11, 12]. However, only one STB course with small sample size equipped with Caesar (a trauma patient simulator) has been reported in China [13]. But, the cost of the trauma simulator is relatively high and difficult to obtain. It is crucial to introduce STB skills courses utilizing proper teaching methods to general Chinese medical students without expensive equipment.

Medical students are the primary target population of STB training courses. Education in the course traditionally includes demonstrations, lectures, and hands-on teaching sessions [14–16]. Although students’ skills can be enhanced through these traditional teaching methods; training teamwork skills are often neglected. It can be difficult for medical students to manage complex clinical scenarios in a real-life trauma setting after completing a course that emphasizes single-skills training and de-emphasizes teamwork-based training. In a traumatic event, responding students are required to make comprehensive decisions in real time, including asking for help, diagnosing injuries, assigning tasks, transferring the patient, implementing clinical interventions, and more [17]. Furthermore, medical training is about acquiring clinical skills and cultivating a state of mind that will allow students to embrace the sacrifice and love for humanity embedded in the Hippocratic oath [18]. These comprehensive abilities should be enhanced through teamwork-based training. To facilitate this learning, a novel problem-, team- and evidence-based learning (PTEBL) approach to teaching may compensate for the weaknesses of traditional teaching methods [19]. We conducted a cluster randomised controlled trial to compare PTEBL teaching approach (intervention) to a traditional course (control) among medical students of a five-year training program from the Xiangya School of Medicine, Central South University.

This research aimed to evaluate the effectiveness of PTEBL, a novel teaching method, via comparison between an experimental PTEBL and control traditional teaching group. It was hypothesized that implementing a PTEBL teaching approach in the STB course could contribute to better teamwork skills and noninferior hemorrhage controlling skills compared with the traditional teaching method.

## Methods

### Study design

This is a parallel group, unmasked, randomized clinical trial (RCT) using online surveys completed before and after the STB course. STB was launched in the situation of increasing number of gunshot injuries in the United States, while there may be more traffic accident injuries in China. Traffic accident injuries usually involve complex injury processes. Therefore, the traffic accident injuries might need team work more. We applied the PTEBL teaching approach to fit the new situation..Students of this case study were randomized into either an experimental group utilizing the PTEBL teaching approach (*n* = 77) or a control group utilizing the traditional teaching approach (*n* = 76), using a 1:1 allocation ratio. Random grouping is mainly achieved through random numbers. Every group was led by a single instructor. The instructor in PTEBL group has experiences in educational reform, and has published related articles on the PTEBL teaching method [19] and STB training [13]. The instructor has also completed a “Stop the Bleed” training certificate. The instructor in the traditional group was trained in China and follows a traditional teaching mode. Both teachers were provided with scripts to follow. They prepared the lessons before each class. Each instructor also engaged students by asking questions to ensure students were learning the technique correctly. In addition, the teaching courses for both student groups had the same teaching duration of four hours on Jun 14, 2022. A 15-min break was provided for every 45 min of class. All courses were completed in the laboratory of the teaching building of Xiangya School of Medicine. Each instructor taught 16 to 17 students per class (teacher to student ratio: 1:16–17). In the questionnaire [20], students were queried about their mastery of STB skills and willingness to apply these skills during a traumatic medical emergency, etc. The questionnaires also included statistics to assess the students’ attitudes of willingness to provide aid to a bleeding patient. The outcomes of the questionnaire were analyzed to assess the effectiveness of the PTEBL teaching approach. (Appendices 1 and 2) The trial was registered in the Central South University (No. 2021JY188). No incentives / reimbursements were provided to participants.

The learner attendance, the materials and the educational strategies used in the educational intervention and the duration for the educational intervention were assessed by raters. Raters were two doctoral level students, trained by senior staff.

### Participants

**Fig. 1:**
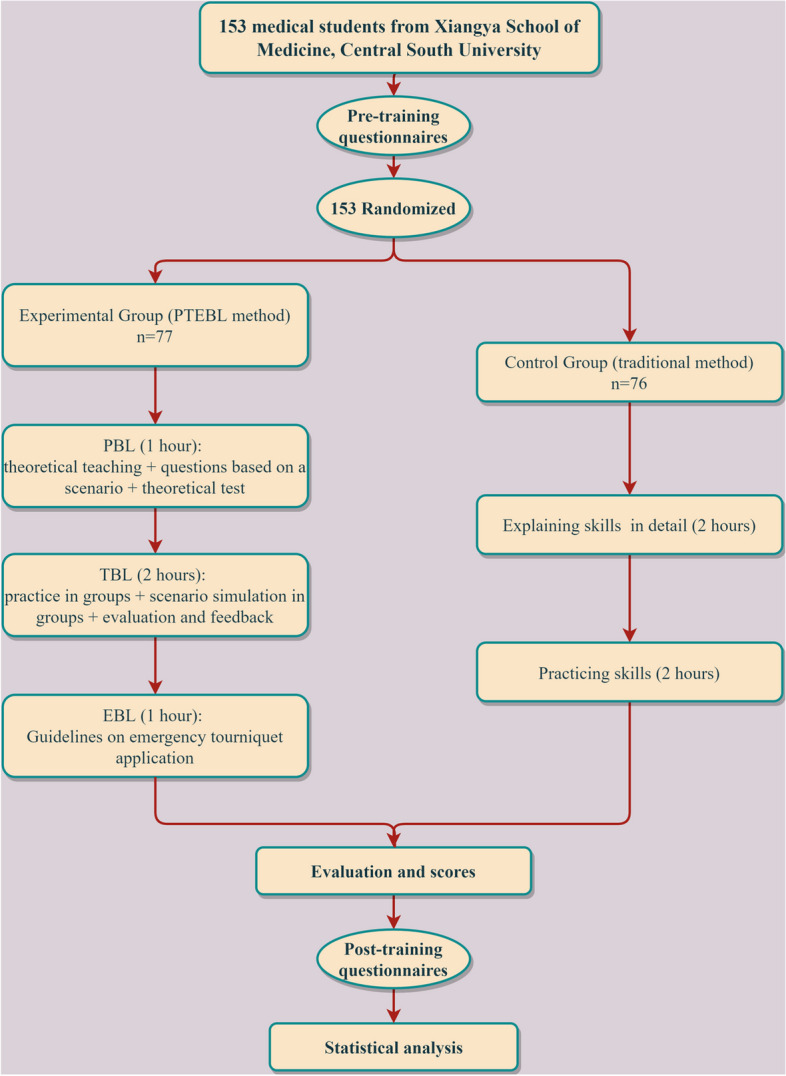
Enrollment, randomization, and protocol of participants

All participants were the third-year medical students of a five-year training program from the Xiangya School of Medicine, Central South University. STB is a course for all people regardless of medical background. However, considering that our medical students still have gaps in hemostatic skills, we intended to incorporate this advanced skill into our training program for medical students. We have released recruitment information on Apr 30, 2022 and included medical students who voluntarily participated in the trial. We excluded students who have received systematic hemostatic training due to certain opportunities. One hundred fifty-three participants were randomized into two study groups (Fig. 1). We generated random numbers using IBM SPSS Statistics v26.0 statistical software. The demographic data of participants in age and sex are shown in Table 1. Informed consent was obtained from all participants enrolled in the study.

**Table 1:** Demographic data of participants

	Overall (*n* = 153)	PTEBL method (*n* = 77)	Traditional method (*n* = 76)	*P* value
Age, mean ± SD	20.6 ± 0.7	20.7 ± 0.8	20.5 ± 0.7	0.489
Female sex	53.6% (82/153)	53.2% (41/77)	53.9% (41/76)	0.931
No experience in stopping the bleed^a^	41.2% (63/153)	45.5% (35/77)	36.8% (28/76)	0.279
Minimal basic training in hemorrhage control techniques^a^	58.8% (90/153)	54.5% (42/77)	63.2% (48/76)	0.279

^a^All students were considered to have no good training experience in hemostasis techniques

### Study protocol

Prior to the course, participants completed both an anonymous pre-training questionnaire about their prior experiences with hemorrhage control techniques and a post-training questionnaire about their confidence levels with applying these techniques after completion of the course. (See Appendix 1 Pre-Questionnaire and Appendix 2 Post-Questionnaire [12, 21]).

For the traditional teaching method, the instructor demonstrated three fundamental skills for obtaining hemostasis (Direct Finger Compression, Packing, and Tourniquet Placement) while describing each step and explaining techniques in detail according to the standard STB (two hours in this part). Students then practiced these three skills for stopping bleeding (two hours in this part). At the end of the course, instructors evaluated and scored each participant’s skill level (Fig. 1).

For the PTEBL teaching approach implemented in the experimental group, classes included three sessions: 1) problem-based learning (PBL) (1.5 h in this part), 2) team-based learning (TBL) (two hours in this part), and 3) evidence-based learning (EBL) (0.5 h in this part). The PTEBL teaching approach emphasized four steps in the EBM process a) developing an answerable question, b) finding the best available evidence, c) evaluation the evidence and d) applying the evidence to a patient care decision.

The first session presented theoretical knowledge and posed questions to students. Students read a scenario of traumatic bleeding adapted from a medical TV series. Instructors then posed four questions about the operation of pre-hospital emergency medical services: (Q1) How can bleeding be stopped effectively? (Q2) When should cardiopulmonary resuscitation be initiated? (Q3) Which actions were performed well? (Q4) Which actions were not performed well? After learning Direct Finger Compression, Packing, and Tourniquet Placement academic knowledge using interactive multimedia, students completed a 3-item knowledge quiz (see Appendix 3 Theoretical Test) to gauge the efficacy of theoretical teaching and the students’ comprehension.

In the second session, participants were divided into small groups to practice hands-on bleeding control skills and to provide critiques to their team members. After instruction with tourniquet placement, where each student had an opportunity to perform at least one placement, each team member played different roles in the scenario simulation: the injured victim, the injured victim's friend, the primary rescuer, and the rescuer’s colleague. The simulation involved a disabled individual sustaining an active brachial artery injury after a ground level fall. After direct finger compression, packing, and tourniquet placement were implemented by the team, bleeding control was achieved. During the simulation, team members made comprehensive decisions through collaboration, including assigning tasks, transferring patients, and implementing emergency medical services. After this scenario, participants described their experiences acting in different roles. Trained STB instructors observing the scenario evaluated their operation and provided participants with feedback on proper hemorrhage control techniques.

In the last session, instructors contributed to establishing competencies for medical students by adhering to expert consensus standards on emergency tourniquet application derived from current International Medical Association guidelines [22–25]. The consensus presented an outline of international guidelines and practices in emergency medicine [26, 27].

### Statistical analysis

Statistical analysis was performed using IBM SPSS Statistics v26.0 statistical software. Continuous variables were expressed as the mean with standard deviation. Categorical variables were defined as frequency and compared using a paired *X*^*2*^ test. Wilcoxon signed-rank test was used for the ordered variables of the Likert scale data. Spearman's correlation coefficient (CC) was applied to analyze the correlation between the variables, and the results were presented as a correlation heatmap. The greater the absolute value of CC, the stronger the correlation. When the absolute value of CC is between 0.9 and 1, variables are highly correlated. When the absolute value of CC is between 0.7 and 0.9, variables are strongly correlated [21]. A *P*-value of < 0.05 was considered statistically significant. The "strongly agree" (5) and "agree" (4) components of the Likert scale were transferred into one, and the remaining three components were changed into zero, namely converting the variables into dichotomous variables for statistical analysis. To estimate the number of samples, an a priori power analysis was performed using G*power v3.1 (UCLA Statistical Consulting Group, Los Angeles, CA) based on repeated measures within *X*^*2*^ tests, with a hypothesized effect size of 0.3, an α error of 0.05 and a power of 0.95, which resulted in a sample size of *n* = 145. Our total sample size is 153, which meet the requirements.

## Results

### Characteristics of participants

A total of 153 participants participated in the study. All participants completed two questionnaires before and after the STB course independently. There were no statistically significant differences (*P* > 0.05) in demographics for the experimental group (*n* = 77) compared with the control group (*n* = 76). Meanwhile, the characteristics of participants in prior basic knowledge of obtaining hemostasis are shown in Table 1. 41.2% (63/153) of the participants had no experience in hemorrhage control techniques; 58.8% (90/153) of the participants only had minimal basic training in hemorrhage control techniques. No record of any specific adaptations made to the educational intervention was kept.

### Mastery level of hemostasis skills

Proficiency of hemostasis skills in compression via direct finger compression, packing, and tourniquet placement before and after the PTEBL and the traditional methods are presented in Table 2. Both the PTEBL method and the traditional method had statistically significant differences (*P* < 0.001) in reported proficiency before and after the course. However, there were no statistically significant differences between the PTEBL method and the traditional method in proficiency of fundamental hemostatic skills (*P* = 0.243, 0.645, and 0.280, respectively). No record of any modifications made during the course of the educational intervention was retained.

**Table 2:** Students' proficiency in different hemostatic skills

	PTEBL method		*P* value	Traditional method		*P* value
	Pre-course	Post-course		Pre-course	Post-course	
Finger compression	44.2% (34/76)	79.2% (61/77)	< 0.001	43.5%(33/76)	76.3%(58/76)	< 0.001
Packing	37.7% (29/77)	93.8% (72/77)	< 0.001	23.7%(18/76)	89.5%(68/76)	< 0.001
Tourniquet placement	33.8% (26/77)	92.2% (71/77)	< 0.001	25.0%(19/76)	88.2%(67/76)	< 0.001

### Rescue attitude

The number of participants who felt prepared to help and those who would refuse to provide assistance in a trauma even pre- and post-course were recorded and shown in Table 3. Again, there were statistically significant differences (*P* < 0.001) in the PTEBL group pre- course and post-course. There were also statistically significant differences (*P* < 0.001) in the traditional group. But no statistically significant differences (*P* > 0.05) between the PTEBL group and the traditional group.

**Table 3:** Rescue attitude in trauma scene

	PTEBL method		*P* value	Traditional method		*P* value
	Pre-course	Post-course		Pre-course	Post-course	
Prefer to help	50.6% (39/77)	96.1% (74/77)	< 0.001	65.8% (50/76)	90.8% (69/76)	< 0.001
Refuse to help	49.4% (38/77)	3.9% (3/77)	< 0.001	34.2% (26/76)	9.2% (7/76)	< 0.001

Effectiveness evaluation of the PTEBL method and the traditional method.

**Fig. 2:**
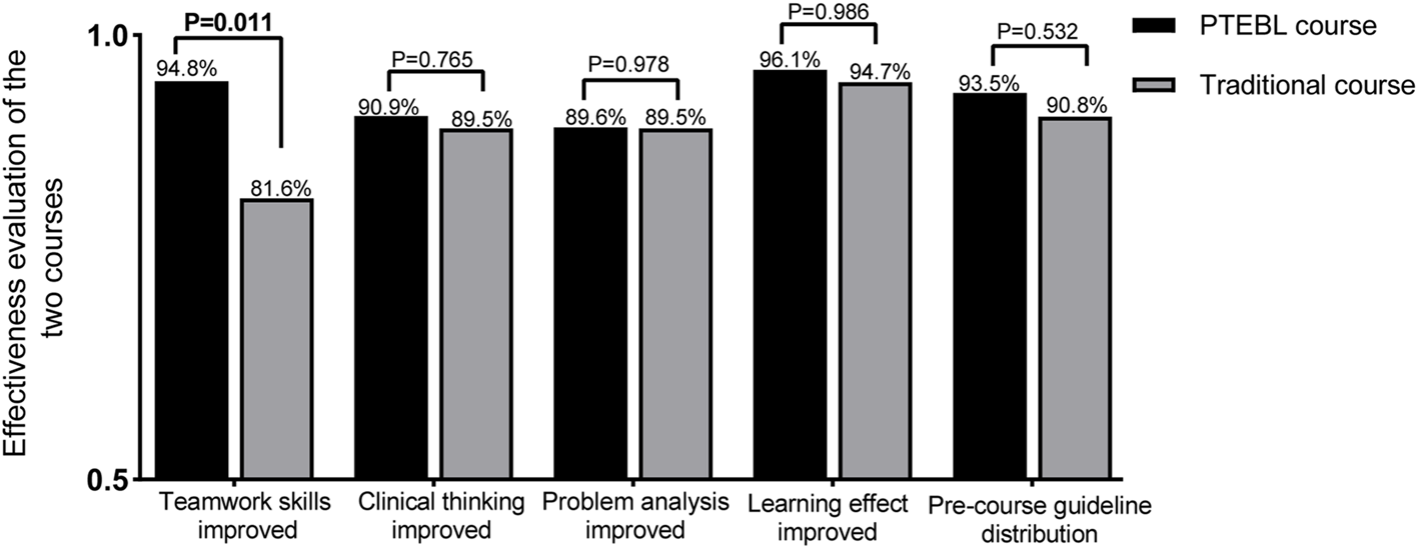
Effectiveness evaluation of the two groups

Evaluation of the effectiveness of the PTEBL method and the traditional method based on five indicators are presented in Fig. 2. 94.8% (73/77) of the PTEBL course participants believed their teamwork skills were improved, while 81.6% (62/76) of the traditional course participants believed their teamwork skills were improved, with a statistically significant difference (*P* < 0.05) observed. There were no statistically significant differences in teaching effectiveness between the PTEBL method and the traditional method (*P* > 0.05).

Performance assessment of the PTEBL method and the traditional method.

There was no statistically significant difference (*P* > 0.05) between the assessment scores after the PTEBL method (92.9 ± 2.8) compared to those after the traditional method (92.9 ± 2.1).

Correlation heatmap of relevant independent variables.

**Fig. 3:**
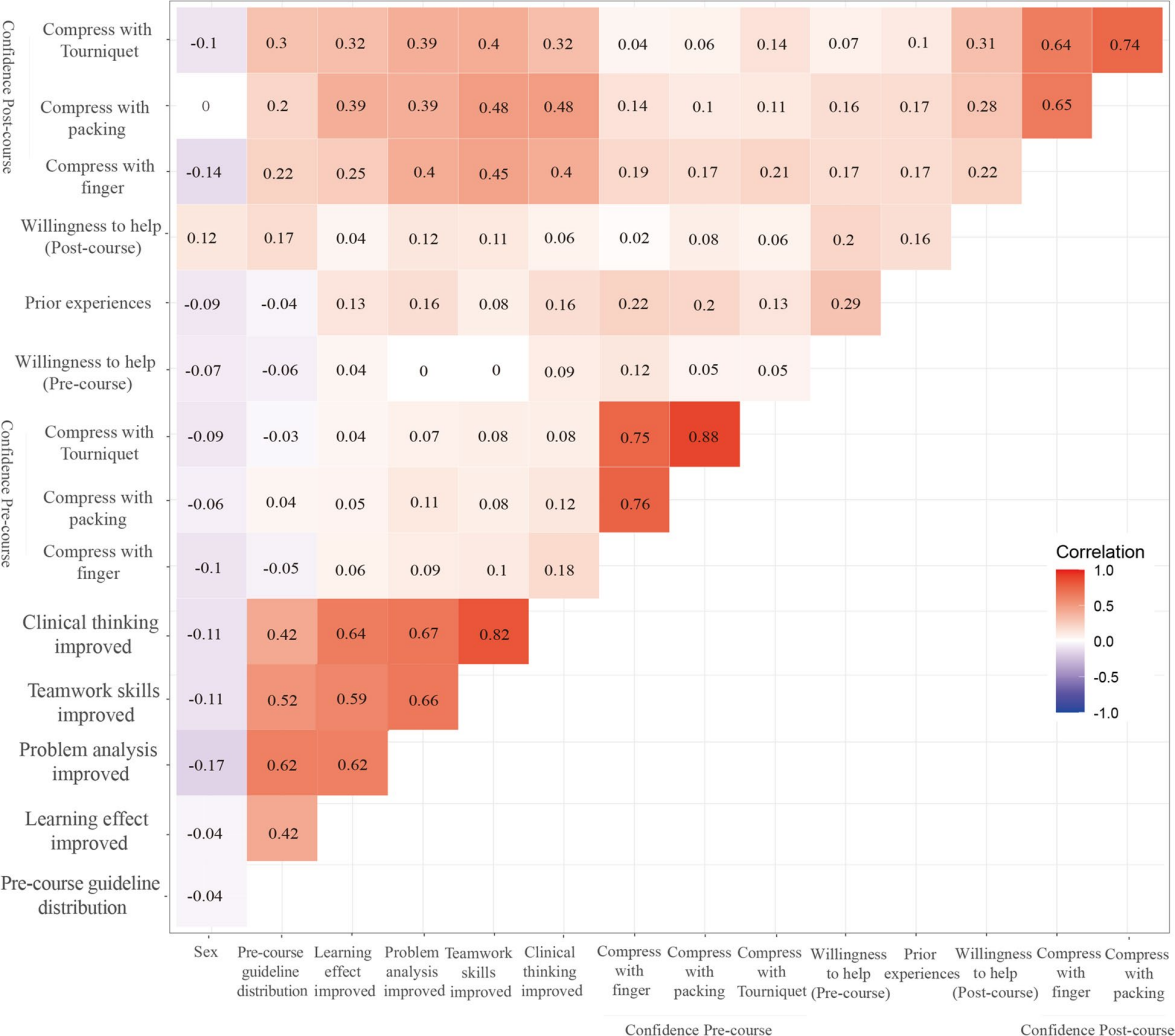
Correlation heatmap of relevant independent variables

The Spearman CC heatmap is shown in Fig. 3. The highest positive correlation in specific skills was observed between pre-course confidence with compression via packing and pre-course confidence with compression via tourniquet placement (*R* = 0.88; 95%CI: 0.81–0.93; *P* < 0.001). The second-highest positive correlation was observed between reported improved clinical thinking and reported improved teamwork skills on the post-course questionnaire (*R* = 0.82; 95%CI: 0.74–0.88; *P* < 0.001). There were three groups of variables with positive correlations of *R* values greater than 0.7 and less than 0.8, which were the correlation between pre-course confidence with compression via direct finger pressure and pre-course confidence with compression via packing (*R* = 0.76; 95%CI: 0.66–0.84; *P* < 0.001), pre-course confidence with compression via direct finger pressure and pre-course confidence with compression via tourniquet placement (*R* = 0.75; 95%CI: 0.66–0.82; *P* < 0.001), and post-course confidence with compression via packing and post-course confidence with compression via tourniquet placement (*R* = 0.74; 95%CI: 0.63–0.83; *P* < 0.001). Other correlations are indicated in Fig. 3.

## Discussion

In summary, our initial hypothesis was confirmed that the application of PTEBL in STB courses contributes to better teamwork. Furthermore, results of our pre-post evaluation demonstrated an increase in bleeding control knowledge, skills and willingness to be first responders regardless of the teaching methods, which indicates the PTEBL method could be applied in the STB courses in China.

These observations are consistent with the results of some prior studies. In a study by Goralnick et al., hemorrhage-control training consisting of a lecture followed by hands-on skills training (87.7% proven to be effective) was found to be the most effective method to enable laypersons to control hemorrhage using a tourniquet [10]. Kaori et al*.* also suggested that STB training lectures with a practical session improved tourniquet knowledge and prepared Japanese citizens for mass casualty events [28]. Generally, the teaching method of “demonstration-practice-examination”, a single skill operation with little teamwork-based teaching, does have remarkable effectiveness, proven by its high utilization in traditional hemostasis training and widespread use in different countries [14, 15]. However, the best form of education for the STB course is still a source of debate [2]. Despite individual hemorrhage-control skills being enhanced through this training; it is also important to note that teamwork, cooperation and, comprehensive ability to respond to emergencies are also important in a trauma scenario.

The novel PTEBL teaching method was first applied in an STB course in China by our group [13]. In the present study without Caesar, students felt more inclined to express their opinions based on problems occurring during a trauma response, and a team-based approach encouraged collaborative thinking. Their abilities to analyze issues independently and think critically were also improved effectively. Furthermore, students worked in teams to practice and simulated clinical scenarios in which different emergency tasks were assigned to every individual. PTEBL achieved an overall improvement in personal and group development, improved the ability of students to integrate skills, especially in terms of communication skills, critical thinking, evidence-based thinking, and successfully prepared students for future clinical work [19]. Findings of Orlas et al*.* have previously supported that via STB course lectures and hands-on skills practice, 92.1% of all participants from different groups felt confident in being able to apply a tourniquet correctly [1]. Our study found that 92.2% of participants in the PTEBL course and 88.2% of participants in the traditional course could successfully apply a tourniquet after training. We suspect that this difference may be due to initial problem-based learning allowing for multiple practice opportunities and real-time feedback to correct mistakes or address overconfidence in some medical students [11].

Given that no statistically significant difference was seen in five other areas besides improved teamwork skills, including clinical thinking, problem analysis, learning effect, performance assessment, and pre-course guideline distribution, we cannot conclude that our new PTEBL teaching method performs better than the former traditional method remarkably. However, we observed that students' team cooperation ability was significantly improved in the PTEBL group compared with the traditional group due to team-based simulation scenarios. The mastery of bleeding control knowledge, skills and willingness to be first responders were also increased after PTEBL method on the basis of data from questionnaires, built on many references [21]. Although the quality control of data may be affected to some extent, students often assess relevant skills and have a more accurate grasp of their skill level and self-confidence, reducing the research error. The heatmap demonstrated improved problem analysis correlated with pre-course guideline distribution, improved learning effect, and improved teamwork skills. Improved clinical thinking is also associated with enhanced learning effects, improved problem analysis, and teamwork skills. Those comprehensive abilities may change reciprocally due to STB training, which suggests that these items influence mutually. In addition, the confidence of hemostasis skills to compress via direct finger pressure, packing, and tourniquet placement also correlate significantly with each other pre- or post-course, suggesting the same principles and techniques of hemostasis were conveyed. Based on our overall results, we believe that PTEBL would be beneficial for developing comprehensive emergency response competence and teamwork skills in particular, and would be superior to traditional methods of teaching STB courses.

A study by Dhillon et al*.* evaluated all participants of an American College of Surgeons STB course and reported a high likelihood of utilizing hemorrhage control skills upon completion of the class, between 95.5%-97.9% [29]. Moreover, the STB protocol has been well received in Italy and rendered good results among civilian health professionals and medical students [30]. In the Middle East, lay members of the public have contributed to a positive response to trauma emergencies after STB training [31]. However, such standardized bleeding control curriculum rely heavily on the acquisition and accessibility of specific equipment and materials, including tourniquets. Thus, the lack of cost and equipment, which would be readily accessible when required, contributed to only a few participants obtaining the necessary materials to mount an appropriate trauma response, as well as the practical education needed for long term use. This suggests that professional tourniquets should be readily available in a public area or commercially in stores, especially in China. Today, AEDs are designed to be simple enough to be used by any individual regardless of training [32]. If we wish for STB courses to have a significant impact on reducing risk of death in a trauma setting, we must create an environment where people can obtain hemostasis tools and materials in case of an emergency. Otherwise, the STB course would be a waste of time, cost, and usefulness.

This study has several limitations. Firstly, the hemorrhage-control ability was measured using self-report questionnaires, which may not accurately reflect practice competence [31]. Secondly, our study analyzed data from a small sample, making it difficult to generalize to other populations comprehensively [33]. The STB project is designed for public education, for which the effect should be evaluated in larger public groups, including all medical personnel and laypersons [5]. The third limitation is that our study only focused on the results of the current course and failed to demonstrate retention of STB skills. However, nearly all STB research has been limited by use of pre- and post-course assessment models [14, 34]. The ideal outcomes measures would be both current and long-term retention after this educational intervention, which would allow improved ability to assess effectiveness of novel teaching methods. The fourth limitation is that the injured victim was all played by group participants rather than real traumatic bleeding patients in our trial, which may lead to a loss of accuracy and scientificity in evaluating student performance. Considering the lack of medical permission and suitable patients, we schedule to test them at the last year of those students (two years after the course) to reveal whether long-term retention after this educational intervention would allow improved ability to cure the real patients. Moreover, building a team in real life is quite difficult, single practice can’t reflect the effectiveness of the PTEBL teaching method and the improvement of teamwork may not necessarily benefit every STB action.

## Conclusions

This study suggests that PTEBL approach increases teamwork during hemorrhage control and manifests superior effectiveness compared with the traditional teaching method. Therefore, the PTEBL deserves to be introduced in STB courses to prevent future deaths from life-threatening hemorrhages in China.

### Supplementary Information

**Supplementary Material 1. **

**Supplementary Material 2. **

**Supplementary Material 3. **

## Data Availability

The datasets used and/or analyzed during the current study are available from the corresponding author on reasonable request.
