# Supplementary material for: A randomized controlled trial comparison of PTEBL and traditional teaching methods in “Stop the Bleed” training
Source: BMC Med Educ. 2024 Apr 26;24:462. doi: 10.1186/s12909-024-05457-4 (PMC11055269; doi:10.1186/s12909-024-05457-4)
Supplement: Supplementary file 1 — Supplementary Material 1. [file 12909_2024_5457_MOESM1_ESM.docx]

**Additional File 1 Pre-Questionnaire**

The following is an optional survey about bleeding and hemorrhage control. Your decision to complete the survey or not and your responses to the questions will have no impact on your grades and evaluations. Nevertheless, we sincerely appreciate your contribution to this work.

1. What is your prior experience with bleeding/hemorrhage control?

○ No experience

○ Minimal basic training in hemorrhage control techniques

○ Formal training in hemorrhage control techniques

○ Have used hemorrhage control techniques on a live person

2. If you witnessed a mass casualty event tomorrow and saw someone with life-threatening femoral artery bleeding from an amputated leg, would you try to control the bleeding?

○ Yes

○ No

○ I don’t know

3. What is your reason(s) for NOT trying to control the bleeding?

○ I am afraid of blood

○ I would just not get involved

○ I am not sure of what to do

○ Something else: ________

4. Have you ever compressed with fingers on a wound to control the bleeding?

○ Yes

○ No

5. Have you ever compressed with packing to control bleeding?

○ Yes

○ No

6. Have you ever compressed with a tourniquet on someone?

○ Yes

○ No

7. How confident are you in the following ability before classes?

(Not at all) 1 2 3 4 5 (very)

Compress with fingers ○ ○ ○ ○ ○

Compress with packing ○ ○ ○ ○ ○

Compress with tourniquet ○ ○ ○ ○ ○

8. How important is it for the following groups of the population to receive formal hemorrhage-control training?

(Not at all) 1 2 3 4 5 (very)

Medical graduates ○ ○ ○ ○ ○

Medical postgraduates ○ ○ ○ ○ ○

Doctors in the hospital ○ ○ ○ ○ ○

Hospital technicians, logisticians, and administrators ○ ○ ○ ○ ○

General public ○ ○ ○ ○ ○

9. How important is it to have hemorrhage-control kits available in public areas, as AEDs are?

(Not at all) 1 2 3 4 5 (very)

○ ○ ○ ○ ○

10. Should formal hemorrhage-control training be incorporated into the medical school curriculum? If so, when?

○ It should not be taught during medical school

○ It should be optional

○ During the 1st year in medical school

○ During the anatomy course

○ During the surgery course

○ During the last year in an internship

○ During the work in the hospital

11. What is the professional field you want to pursue in the future?

○ Internal Medicine

○ Surgery Medicine

○ Acute and Critical Care Medicine

○ Other Specialties

○ None-medical industry

○ I don’t know
