# Supplementary material for: A randomized controlled trial comparison of PTEBL and traditional teaching methods in “Stop the Bleed” training
Source: BMC Med Educ. 2024 Apr 26;24:462. doi: 10.1186/s12909-024-05457-4 (PMC11055269; doi:10.1186/s12909-024-05457-4)
Supplement: Supplementary file 2 — Supplementary Material 2. [file 12909_2024_5457_MOESM2_ESM.docx]

**Additional File 2 Post-Questionnaire**

The following is an optional survey about bleeding and hemorrhage control. Your decision to complete the survey or not and your responses to the questions will have no impact on your grades and evaluations. We sincerely appreciate your contribution to this work.

1. Now that you have received hemorrhage-control training, if you witnessed a mass casualty event tomorrow and saw someone with life-threatening femoral artery hemorrhage from an amputated leg, would you try to control the bleeding?

○ Yes

○ No

○ I don’t know

2. What is your reason(s) for NOT trying to control the bleeding?

○ I am afraid of blood

○ I would just not get involved

○ I am not sure of what to do

○ Something else: ________

3. Now that you have received hemorrhage-control training, how confident are you in the following ability?

(No) 1 2 3 4 5 (Yes)

Compress with fingers ○ ○ ○ ○ ○

Compress with packing ○ ○ ○ ○ ○

Compress with tourniquet ○ ○ ○ ○ ○

4. How important is it for the following groups of the population to receive formal hemorrhage-control training?

(Not at all) 1 2 3 4 5 (very)

Medical graduates ○ ○ ○ ○ ○

Medical postgraduates ○ ○ ○ ○ ○

Doctors in the hospital ○ ○ ○ ○ ○

Hospital technicians, logisticians and administrators ○ ○ ○ ○ ○

General public ○ ○ ○ ○ ○

5. How important is it to have hemorrhage-control kits available in public areas, as AEDs are?

(Not at all) 1 2 3 4 5 (very)

○ ○ ○ ○ ○

6. Should formal hemorrhage-control training be incorporated into the medical school curriculum? If so, when?

○ It should not be taught during medical school

○ It should be optional

○ During the 1st year in medical school

○ During the anatomy course

○ During the surgery course

○ During the last year in an internship

○ During the work in the hospital

7. What is the professional field you want to pursue in the future?

○ Internal Medicine

○ Surgery Medicine

○ Acute and Critical Care Medicine

○ Other specialties

○ None-medical industry

○ I don’t know

8. Do you have any interest in becoming a Stop the Bleed instructor?

○ Yes

○ No

9. After this training, do you feel yourself

(Strongly disagree) 1 2 3 4 5 (Strongly agree)

Teamwork skills were improved ○ ○ ○ ○ ○

Clinical thinking was improved ○ ○ ○ ○ ○

Problem analysis was improved ○ ○ ○ ○ ○

Scenario simulation enhanced learning ○ ○ ○ ○ ○

The distributed PowerPoint and study resources were helpful for learning

○ ○ ○ ○ ○

10. For this training, what do you think of the teacher?

(Very dissatisfied) 1 2 3 4 5 (Very satisfied)

Enthusiasm for lectures ○ ○ ○ ○ ○
Interactivity with students ○ ○ ○ ○ ○

Overall teaching effect ○ ○ ○ ○ ○

11. What would you suggest for the course? Thanks for providing valuable advice.

______________________________________________
