# Supplementary material for: A randomized controlled trial comparison of PTEBL and traditional teaching methods in “Stop the Bleed” training
Source: BMC Med Educ. 2024 Apr 26;24:462. doi: 10.1186/s12909-024-05457-4 (PMC11055269; doi:10.1186/s12909-024-05457-4)
Supplement: Supplementary file 3 — Supplementary Material 3. [file 12909_2024_5457_MOESM3_ESM.docx]

**Theoretical Test**

1. When using a tourniquet to stop bleeding, what are the details to pay attention to?
2. What are the meanings of ‘ABCDEF’ in trauma physical examination and ‘CRASH-PLAN’ in secondary assessment?
3. What is the meaning of ‘ABC’ in trauma hemostasis?
